# Metallomic mapping of gut and brain in heavy metal exposed earthworms: A novel paradigm in ecotoxicology

**DOI:** 10.1016/j.bbrc.2024.149827

**Published:** 2024-05-21

**Authors:** Maxim A. Karpov, Carl Hobbs, Suwan N. Jayasinghe, Stephen R. Stürzenbaum

**Affiliations:** a Department of Analytical, Environmental and Forensic Sciences, Faculty of Life Sciences and Medicine, King's College London, London, United Kingdom; b Wolfson Sensory Pain and Regeneration Centre, King's College London, London, United Kingdom; c Department of Mechanical Engineering, BioPhysics Group, University College London, United Kingdom

**Keywords:** Earthworm, Chloragogenous tissues, Lead bioaccumulation, Soil analysis, Ecotoxicology, LA-ICP-MS

## Abstract

This study explored the uptake of lead in the epigeic earthworm *Dendrobaena veneta* exposed to 0, 1000, and 2500 μg Pb/g soil. The soil metal content was extracted using strong acid digestion and water leaching, and analysed by means of Inductively Coupled Plasma Mass Spectrometry (ICP-MS) to estimate absolute and bioavailable concentrations of metals in the soil. The guts and heads of lead-exposed earthworms were processed into formalin-fixed and paraffin embedded sections for high-resolution multi-element metallomic imaging via Laser Ablation ICP-MS (LA-ICP-MS). Metallomic maps of phosphorus, zinc, and lead were produced at 15-μm resolution in the head and gut of *D. veneta*. Additional 4-μm resolution metallomic maps of the earthworm brains were taken, revealing the detailed localisation of metals in the brain. The Pb bioaccumulated in the chloragogenous tissues of the earthworm in a dose-dependent manner, making it possible to track the extent of soil contamination. The bioaccumulation of P and Zn in earthworm tissues was independent of Pb exposure concentration. This approach demonstrates the utility of LA-ICP-MS as a powerful approach for ecotoxicology and environmental risk assessments.

## Highlights

• Lead exposure of earthworms causes a dose-dependent accumulation of Pb in the chloragog.
• The distribution of P and Zn in earthworm tissues is independent of Pb exposure dose.
• Metallomic LA-ICP-MS imaging promises to be a valuable tool for unravelling the detoxification mechanisms in soil invertebrates.

## Abbreviations

FFPE: Formalin Fixed Paraffin Embedded
XRF: X-ray Fluorescence
ICP-MS: Inductively Coupled Plasma Mass Spectrometry
LA-ICP-MS: Laser Ablation Inductively Coupled Plasma Mass Spectrometry

## 1 Introduction

The average concentration of lead in UK soils is around 200 μg Pb/g soil, however, the macro- and micro-scale geological composition as well as the proximity of anthropogenic activities may increase Pb levels by multiple orders of magnitude, and way above safe limits for wildlife and humans [1,2]. Lead is not essential to life and exhibits its toxicity through mimicry of essential metal(loid)s. Specifically, lead tends to dysfunctionally mimic calcium, an important mineral participating in bone and tooth formation, cell signalling, and neuronal signal transduction [3]. This perturbation of endogenous metallome is non-specific but often concerns the neural and muscular systems [4]. The use of lead has therefore been restricted starting with the discontinuation of leaded gasoline in 1990s and the removal of lead from paints [5]. As with other xenobiotic heavy metals, lead bioaccumulates during chronic exposure due to its long half-life and poor rate of elimination. Organisms have evolved systems for metal homeostasis in terms of metal selectivity, transport, and sometimes, metal filtration. However, as evidenced by the deleterious effects of Pb on biological systems, these systems have their limits.

Earthworms are soil-dwelling ecosystem engineers implicated in improving soil fertility by maintaining soil aeration, drainage, and porosity, and increasing nutrient availability to plants, via a myriad of natural lifestyle behaviours [6,7]. As such, these organisms have been extensively used as indicators of soil quality in environmental risk assessments and ecotoxicology [8,9]. Multiple soil quality metrics related to earthworms exist, such as earthworm density, cocoon production rate, gene expression levels, toxicant (e.g. heavy metal) body load, metabolic biomarker levels, however, these metrics can be elusive often due to the evolutionary adaptation of earthworms to the toxicity of local environment [[10], [11], [12], [13], [14], [15]]. For example, communities of earthworms exist in soils of abandoned mine sites such as Cwmystwyth, where soil Pb concentration exceeds the known 2-week LC_50_ for lead [16,17]. Earthworms possess a specialised layer of tissue enveloping their intestines named the chloragogenous tissue which, analogously to liver, is thought to deal with toxic entities consumed by earthworms, including viruses, synthetic compounds, and/or xenobiotic metals [[18], [19], [20], [21]]. In terms of heavy metals, earthworms segregate and seclude toxic particles in membrane bound organelles, termed chloragosomes, which are then stored or possibly excreted through the nephridia or the gut lumen. To date, the details of this detoxification pathway remain elusive, however, the mechanism is known to be specific to the element type, with defined elemental partitioning into different types of chloragosomes [21]. For example, in lead hyper-resistant earthworms, the majority of chloragosomes concentrate calcium in a complex with phosphorus such as the inorganic CaHPO_4_ or organic R–OPO_3_Ca; Ca is thought to be exchanged for Pb, immobilising the lead in a phosphate complex, and causing precipitation [22,23]. Whereas in case of cadmium, the mechanism is largely thought to involve metallothionein, with cadmium partitioning into the sulfur-rich “cadmosomes”, where sulfur is thought to be the cadmium-coordinating part of cysteine residues on metallothioneins [14]. Although metallothionein is primarily a zinc binding protein, zinc was documented to coordinate with elements from both, the lead and the cadmium-accumulating chloragosomes [28].

Metallomic studies in earthworms have yet to exploit the advances in Laser Ablation Inductively Coupled Plasma Mass Spectrometry (LA-ICP-MS) technology to its full potential. Recently, LA-ICP-MS was used to study cadmium accumulation in the earthworm brain and silver nanoparticle accumulation in the earthworm gut, however, due to the resolution of the metallomic scans it was not to provide precise tissue morphology [24]. Moreover, the majority of metallomic studies have focused on *Lumbricus rubellus* or *Eisenia fetida*. This present study explored high resolution metallomics utilizing another epigeic earthworm, *Dendrobaena veneta,* focusing on the head and gut sections of lead exposed worms to simulate a two-compartment model of toxicity. This work contributes to the existing knowledge of earthworm metallomics for applications in ecotoxicology, environmental risk assessment, soil vermiremediation, and even biotechnology (i.e. earthworm quantum dot biosynthesis) [25].

## 2 Materials and methods

### 2.1 Earthworm sourcing, maintenance and exposures

The *Dendrobaena veneta* earthworms designated for the exposure experiments were sourced from a commercial supplier (Yorkshire Worms Ltd.) and maintained in the soil provided by the same supplier. The earthworms were stored in perforated plastic boxes containing moistened native soil, inside an incubator (Sanyo MIR-154) at 15 °C. The soil was moistened with water once a week during the duration of the earthworm storage. Adult clitellate earthworms were used for all experiments.

Air-dried commercial soil (Yorkshire Worms) was thoroughly mixed with a lead nitrate (Fisher Scientific) solution up to the desired soil lead concentration (1000 or 2500 μg Pb/g soil). The *D. veneta* earthworms were transferred into perforated plastic boxes and exposed in an incubator for 2 weeks. Prior to processing, the worms were washed with distilled water and placed on a moist filter paper (Whatman) in a Petri dish for 48 h to void the gut contents. The filter paper was changed once a day to prevent re-ingestion of expelled soil.

### 2.2 Soil analysis

Soil samples (0.5 g dry soil) were digested in 10 mL of Optima grade concentrated HNO_3_ (68% w/w; Fisher Scientific trace metal grade acids) using the Milestone UltraWAVE microwave digestion system (15 min at 220 °C and 110 bar). Digests were diluted by a factor of 50 with purified water to achieve a final HNO_3_ concentration of 0.3 M and spiked with the internal standards to a final concentration of 50 μg/L. For water digestion, 0.5 g of dry soil (in 10 mL of purified water) was mixed in a shaker for 24 h, then centrifuged and the supernatant spiked with the internal standards and Optima grade concentrated HNO_3_ (68% w/w; Fisher Scientific trace metal grade acids) to achieve a final concentration of 50 μg/L and 0.3 M respectively.

All measurements were conducted on a PerkinElmer NexION 350D Inductively Coupled Plasma Quadrupole Mass Spectrometer (ICP-QMS) under Kinetic Energy Discrimination (KED) mode at the London Metallomics Facility, King's College London. The introduction system to the instrument was a Cetac ASX-100 autosampler coupled to a SeaSpray glass nebulizer fitted to a quartz cyclonic spray chamber. Instrument settings and model parameters can be viewed in Supplementary Tables 1 and 2 Quality control was ensured through repeat measurements of acid blanks, a calibrant and a certified reference material from High Purity Standards (CRM-TMDW-500). Analyte measurements were normalized to the internal Ga standard to account for instrument drift and matrix effects, and measurements were subsequently blank corrected by removing the average analyte intensity of repeat blank measurements. The corrected isotopic intensity was converted to concentration measurements by interpolation of neighbouring calibrants. The quality of the interpolation was confirmed by verifying the linearity of the calibration curve.

### 2.3 Surgical procedures

The earthworms were anaesthetised by submersion in cold (4 °C) carbonated water for approximately 8 min to shut down any contractile reflexive function. Anaesthetised earthworms were placed on a dissection mat under a light microscope. The “head” samples were generated by cutting the first six anterior segments at the segmental line using stainless-steel surgical scissors. The “gut” samples were generated by cutting off six segments at the midline between the clitellum and the posterior end.

### 2.4 Tissue sectioning

The decapitated heads of the earthworms were placed in 1 mL of 4% buffered formalin, pH 6.9 (Merck). The fixative was replaced after 5 min and the samples were further fixed for 48 h at room temperature under gentle agitation. The fixed samples were washed in distilled water and dehydrated in 70% ethanol (Merck) for 1 h. Subsequent steps were performed using an automated tissue processor (TP1020, Leica), involving the gradual dehydration in 90% Industrial Methylated Spirit (IMS, Fisher) for 2 h and in 100% IMS for 2 h. The ethanol was removed by submerging the samples (1:1 ratio of IMS to xylene) for 60 h and washed with 100% xylene afterwards for 2 h. Xylene was removed and the tissue was placed in molten paraffin wax for 12 h.

The sample was processed in embedding station (Leica EG 1150H) and the resultant solid wax block trimmed carefully around the area containing the tissue sample, leaving a trapezium-shaped wax stump. The embedded sample was secured on a microtome (Reichert-Jung, Mod. 1140/Autocut) and 7 μm-thick sections of the earthworm tissue were cut and placed in a 42 °C wax section mounting bath (Barnstead Electrothermal). The sections were mounted on microscope glass slides (Fisher) and allowed to dry at room temperature for 3 h in a 60 °C oven. Dried slides were stored at room temperature for future procedures.

### 2.5 Laser Ablation inductively coupled mass spectrometry

For LA-ICP-MS, an Analyte Excite 193 nm ArF*excimer-based LA system (Teledyne Photon Machines, Bozeman, MT, USA) was used, equipped with the HelEx II two-volume ablation cell. The LA system was coupled to a Thermo Fisher Scientific iCAP TQ ICP-mass spectrometer (Thermo Fisher Scientific, Waltham, MA, USA) via the Aerosol Rapid Introduction System (ARIS). Tuning of the instrument settings was performed using a NIST SRM 612 glass certified reference material (National Institute for Standards and Technology, Gaithersburg, MD, USA). LA-ICP-MS images were acquired in a fixed dosage mode, using a circular spot size of 4 and 15 μm.

Imaging was performed on paraffin embedded earthworm brain and gut sections. The samples were dewaxed in xylene for 5 min 3 times, followed by 3 X 5-min 100% ethanol washes. Samples were mounted inside a HelEx II two-volume ablation cell (Teledyne Photon Machines). To correct for instrumental drift, a series of NIST 612 standard ablation scans were performed before and after each section. ICP-MS and positional data were reconstructed using the HDF-based Image Processing software (HDIP, Teledyne Photon Machines Inc., Bozeman, MT, USA). Instrument parameters can be viewed in Supplementary Tables 3 and 4 A bespoke pipeline, written in Python (version 3.8), was used to generate elemental images from reconstructed data and statistics. Negative values, attributed to instrumental noise, were replaced with zeros.

## 3 Results and discussion

**Fig. 1:**
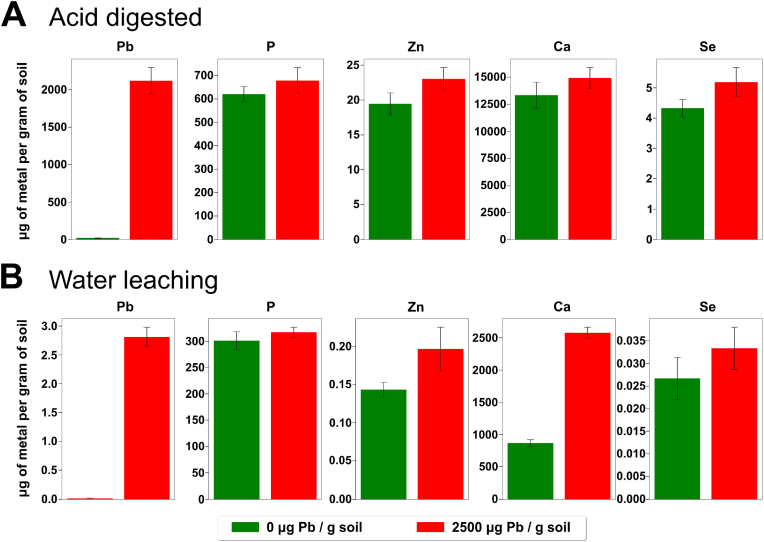
**Soil concentrations of biologically relevant metals extracted via acid digestion or via water leaching.** Metals (lead (Pb), phosphorus (P), zinc (Zn), calcium (Ca), selenium (Se)) were extracted from the soils used in the Pb exposure experiment (green = 0 μg Pb/g, red = 2500 μg Pb/g soil) via **A)** acid digestion or **B)** water leaching, and subsequently quantified by means of Inductively Coupled Plasma Mass Spectrometry. (For interpretation of the references to colour in this figure legend, the reader is referred to the Web version of this article.)

The *Dendrobaena veneta* earthworms and soil were sourced from a UK-based company (Yorkshire worms Ltd). *D. veneta* worms were incubated at 15 °C for 2 weeks in the supplied soil spiked with 0, 1000, and 2500 μg Pb/g soil. This concentration range represented a dose-response, reaching an LC_50_ at 2500 μg/g [16]. After the exposures, the metal composition of the soils was determined by Inductively Coupled Plasma Mass Spectrometry. To estimate the absolute and bioavailable fractions of the metals in the soil, the soil sample preparation protocol included two different types of metal extractions, namely hot nitric acid digestion, and water leaching. The concentrations (μg metal per g of soil) of the biologically relevant metals (Pb, Ca, Zn, P), as well as elements of toxicological and biotechnological interest – such as Cd, Te, Se, were measured in unspiked soil and soil spiked with 2500 μg Pb/g soil (Fig. 1). The levels of tellurium and cadmium were below the instrument detection threshold, thus were not displayed.

Acid digestion data depicts an estimate of the absolute value of metals in the soil. Only 20 ± 2 μg Pb/g soil were detected in the unmodified soil and a concentration of 2120 ± 177 μg Pb/g soil was observed after spiking with lead (II) nitrate. The discrepancy between the spiked amount and the concentration detected could be attributed to the immobilisation of the added lead on soil matrix or the incomplete soil digestion. Irrespectively, this demonstrated that the sourced soil was innately low in toxic heavy metals such as lead and cadmium, hence the *D. veneta* originating from this soil would not be adapted to extreme heavy metal stress. The water leaching experiment, depicting estimates of the bioavailable concentrations of metals in the soil, did not reveal any Pb in the unspiked sample and only 3 ± 0 μg Pb/g soil was observed in the spiked sample. This suggests that the water extraction may underestimate the bioavailable fraction of certain metals such as Pb and Zn. The soil was highly calciferous, which could contribute to the alleviation of lead toxicity. The addition of Pb generally increased the concentrations of metals in the soil. This is likely due to the competition of Pb with other metals for binding sites within the soil matrix.

**Fig. 2:**
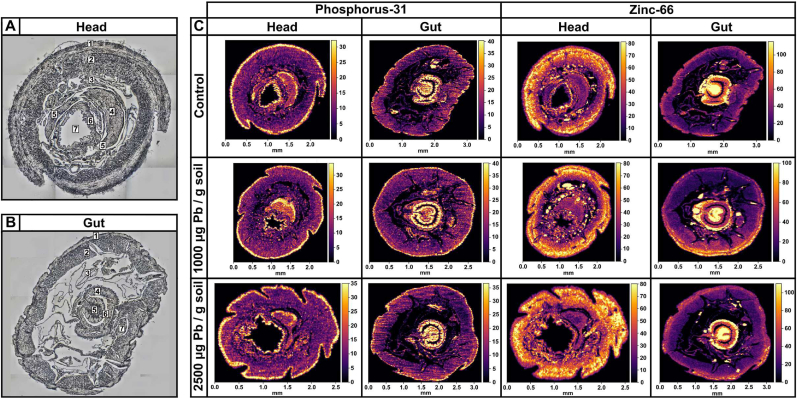
**Elemental maps of phosphorus (**^**31**^**P) and zinc (**^**66**^**Zn) in the gut and head cross-sections of worms exposed to Pb-spiked soil and respective controls.** Cross-section depiction of anatomical features in the (**A**) head and (**B**) gut of *Dendrobaena veneta*. In (**A**) the head section, the numbers correspond to: 1 – epidermis, 2 – muscle, 3 – coelom, 4 – cerebral ganglia, 5 – circumpharyngeal connectives, 6 – pharynx, 7 – pharyngeal lumen. In (**B**) the gut section, the numbers correspond to: 1 – epidermis, 2 – muscle, 3 – coelom, 4 – intestinal wall, 5 – chloragog in typhlosole, 6 – intestinal cavity, 7 – ventral nerve cord. (**C**) Laser Ablation Inductively Coupled Plasma Mass Spectrometry measurements of phosphorus and zinc in the gut and head of formalin fixed paraffin embedded cross-sections of *Dendrobaena veneta* worms at 15-μm resolution. The relative counts per second values are displayed as a colour bar. (For interpretation of the references to colour in this figure legend, the reader is referred to the Web version of this article.)

**Fig. 3:**
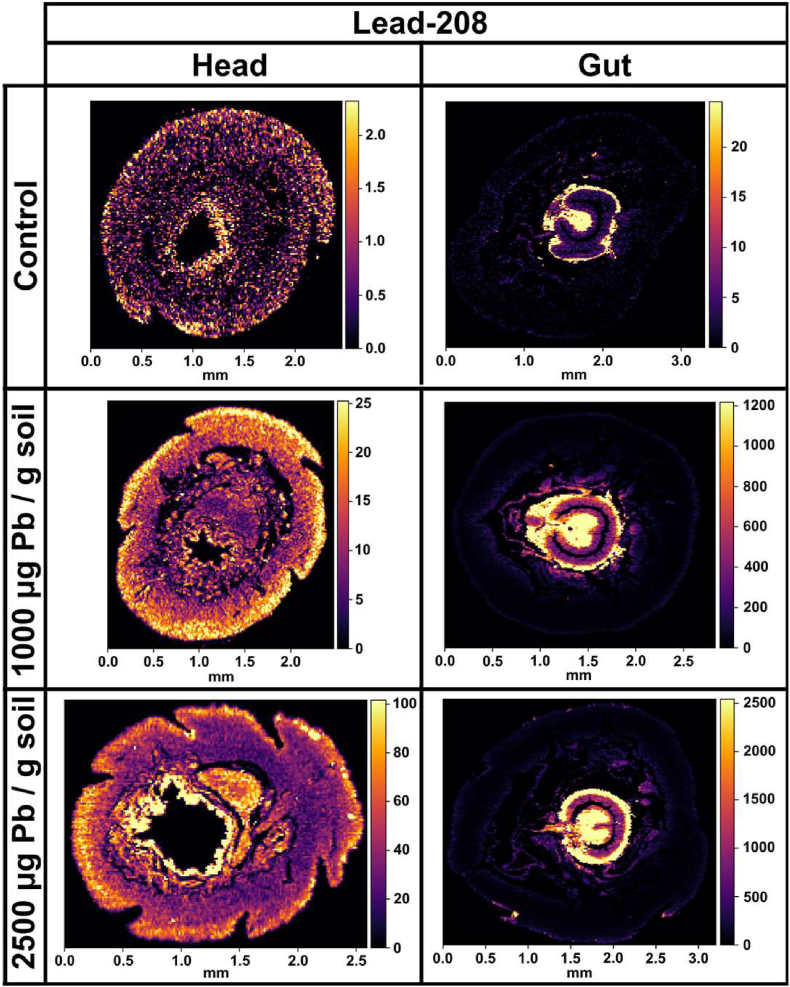
**Elemental maps of lead (**^**208**^**Pb) in the gut and head cross-sections of worms exposed to Pb-spiked soil and respective controls.** Laser Ablation Inductively Coupled Plasma Mass Spectrometry measurements of lead in the gut and head of formalin fixed paraffin embedded cross-sections of *Dendrobaena veneta* worms at 15-μm resolution. The relative counts per second values are displayed as a colour bar. (For interpretation of the references to colour in this figure legend, the reader is referred to the Web version of this article.)

**Fig. 4:**
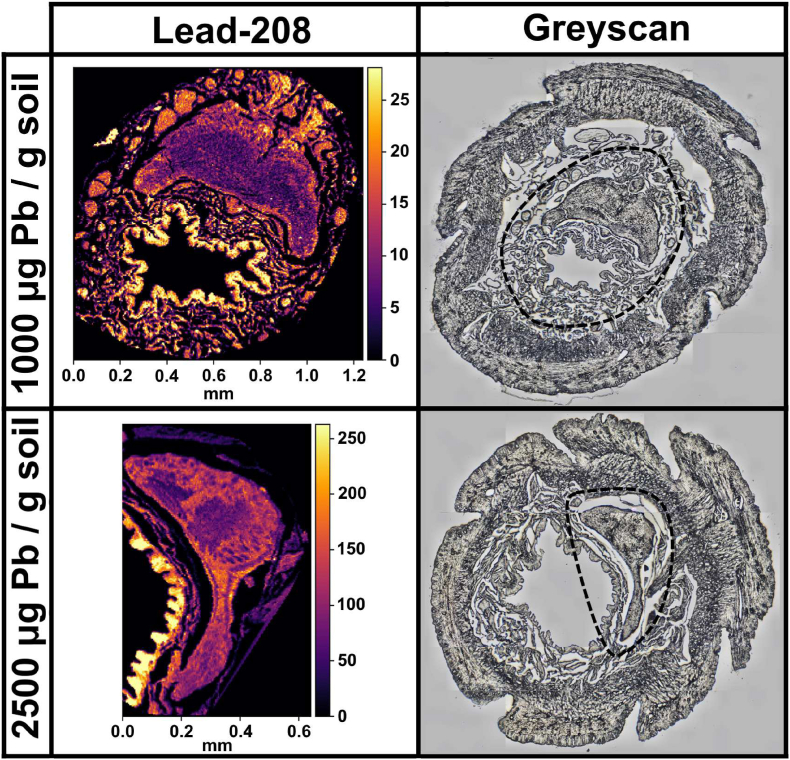
**High resolution magnified elemental maps of lead (**^**208**^**Pb) in the brain of worms exposed to Pb-spiked soil.** Laser Ablation Inductively Coupled Plasma Mass Spectrometry measurements of lead in the region around the cerebral ganglia of formalin fixed paraffin embedded cross-sections of *Dendrobaena veneta* worms at 4-μm pixel resolution. The ablated region has been encircled with a dashed line on the greyscans. A 4-μm image of unexposed control worms was not taken due to the Pb concentrations being under the instrument detection threshold. The relative counts per second values are displayed as a colour bar. (For interpretation of the references to colour in this figure legend, the reader is referred to the Web version of this article.)

Following the Pb exposure, the earthworms were maintained in a Petri dish on a wet filter paper for 2 days to void the gut contents. The specimens were subsequently fixed in formalin and embedded in paraffin (FFPE), before being sectioned in the brain and gut regions. The FFPE sections with an intact morphology were chosen for the Laser Ablation ICP-MS analysis at 15-μm pixel resolution. Mass channels for phosphorus and zinc (Fig. 2), and lead (Fig. 3) facilitated the metallomic quantification of the respective elements. A sequential brain section was reserved and used for further LA-ICP-MS at 4-μm resolution of the cerebral ganglia (Fig. 4). No standards were employed, meaning that the spectrometric measurements represent relative distribution of the chosen metals in the tissue for qualitative comparison.

Phosphorus levels were measured in the gut and head cross-sections (Fig. 2). All head and gut metallomic maps displayed similar concentrations and distributions of phosphorus, which were independent of lead exposure concentration. In the gut sections, phosphorus counts per second peaked in the intestinal region and in the chloragogenous tissues. Because phosphorus is a biologically compatible element, its sequestration in the gut suggests that *D. veneta* may use phosphorus as a component of homeostatic and possibly detoxification pathways. In the brain sections, phosphorus accumulated predominantly in the epidermis and the dorsal side of the cerebral ganglion, where the cell bodies are found. The high phosphorus concentration may reflect a signature of myelination in the earthworm brain, as previously seen from the X-ray elemental microanalysis of rat sciatic nerve [26]. In both tissue section types, phosphorus also hyperaccumulated in the epidermis of the earthworm.

The elemental maps of zinc in the gut cross-sections bioaccumulated in the intestinal walls and the chloragogenous tissues (Fig. 2). Chloragogenous tissues are known to be the key organ participating in the metal homeostasis of the earthworm. It is likely that *D. veneta* regulates zinc availability at the chloragogenous tissue layer to prevent toxicity and malnutrition. In the head region, zinc accumulated sparsely in a diffusive manner from the epidermis towards the worm intestines. Zn concentrations were relatively low in the cerebral ganglia. Interestingly, circular pockets of concentrated zinc were found to be scattered around the intersection between the intestines and the epidermis, possibly pertaining to fluid carrier vessels or brown bodies. Similar to phosphorus, head and gut tissue zinc concentrations were not affected by the exposures to lead.

Finally, the distribution of lead was measured in the gut and head cross-sections of the earthworm (Fig. 3). The gut, in particular the intestines and the chloragogenous tissues, were by far, the main locations of Pb accumulation. In attempt to combat Pb toxicity, the earthworm seems to compartmentalize, and therefore immobilise, the metal in its chloragosomes. The exposed worms accumulated Pb in a dose responsive manner, as seen by the change in cps values (e.g. gut, control: 0–25 cps; 1000 μg Pb: 0–1200 cps; 2500 μg Pb: 0–2500 cps). Despite the high levels of Pb in the gut, the worms did not hyperaccumulate Pb in the head to the same degree, demonstrating that the chloragog serves as a heavy metal sponge, and a defensive barrier for critical organs. The route of Pb uptake is known to be predominantly dermal, possibly through the nephridia pores [29]. However, the worm exposed to 2500 μg Pb/g soil accumulated significantly more Pb in the head than the worm exposed to 1000 μg Pb/g soil, signifying that the chloragogenous filtration system was overburdened, presenting a two-compartment model of Pb bioaccumulation. In general, the spatial pattern of Pb accumulation in the head was sparse and disordered, which suggests the absence of detoxification and lead resistance mechanisms beyond the chloragog. This was exemplified by the 4-μm resolutions brain scans (Fig. 4). The lead was primarily sequestered in the cerebral ganglia of worms exposed to 2500 μg Pb/g soil. This supports the notion that nerve tissues are a primary target of Pb toxicity due to high calcium content.

This paper serves as a preview into the future of high-resolution spatial mapping of metals in the earthworm and further optimization may enhance the protocol. For example, the FFPE process may cause leaching of metals which in turn can affect the biological accuracy of elemental maps. This can be circumvented via cryosectioning, albeit with detriment to the morphological quality of sections, or via laser sectioning [27]. Having said that, the observation that zinc and phosphorus accumulation is independent of lead exposure supports the hypothesis that the earthworm activates a unique lead detoxification pathway, as previously suggested by the X-ray absorption spectroscopy data [22,23]. The presence of such detoxification pathways can now be attributed to *Dendrobaena veneta* inhabiting ecologically clean soils, suggesting that these mechanisms may be universal to all epigeic earthworms. The question of whether the Pb-containing chloragosomes are excreted by the earthworm and the routes of excretion along the longitudinal axis of the earthworm body remain unanswered. LA-ICP-MS is therefore a powerful tool that will allow us to understand the underlying evolutionary adaptation that bestows resistance of earthworms to toxic heavy metal, including the hyper-resistance of individuals inhabiting abandoned lead mine sites, such as Cwmystwyth [17].

## Funding

This work was supported by a 10.13039/501100000268Biotechnology and Biological Sciences Research Council (BBSRC) London Interdisciplinary Biosciences Consortium (LIDo) Doctoral training award (project Reference 2325447) with additional support provided via funding from the 10.13039/501100000270Natural Environment Research Council (NERC) *Environmental* '*Omics Synthesis* Centre (*EOS*).

## CRediT authorship contribution statement

**Maxim A. Karpov:** Conceptualization, Data curation, Formal analysis, Investigation, Methodology, Validation, Visualization, Writing – original draft. **Carl Hobbs:** Investigation, Resources. **Suwan N. Jayasinghe:** Funding acquisition, Resources, Supervision. **Stephen R. Stürzenbaum:** Conceptualization, Funding acquisition, Methodology, Project administration, Resources, Supervision, Validation, Writing – review & editing.

## Declaration of competing interest

The authors declare that they have no known competing financial interests or personal relationships that could have appeared to influence the work reported in this paper.
