# Supplementary material for: Metallomic mapping of gut and brain in heavy metal exposed earthworms: A novel paradigm in ecotoxicology
Source: Biochem Biophys Res Commun. 2024 May 21;709:None. doi: 10.1016/j.bbrc.2024.149827 (PMC11458488; doi:10.1016/j.bbrc.2024.149827)
Supplement: Multimedia component 1 [file mmc1.docx]

**Supplementary materials**

**Supplementary Table 1:** Perkin Elmer NexION 350D ICP Mass Spectrometer Settings.

| **Ar Gas Flow (L min^-1^)** | 1 |
| --- | --- |
| **Ar Auxiliary Gas Flow (L min^-1^)** | 1.2 |
| **Ar Plasma Flow (L min^-1^)** | 18 |
| **Cell A Gas Flow (NH_3_, mL min^-1^)** | 0 |
| **Cell A Gas Flow (He, mL min^-1^)** | 4.5 |
| **Nebulizer Gas Flow (L min^-1^)** | 0.95 |
| **Mode of Operation** | KED |
| **RF Power (W)** | 1600 |

**Supplementary Table 2:** ICP-MS method parameters for soil metal quantification of P, Ca, Zn, Se, Cd, Te, and Pb.

| **Element Mass** | ^31^P, ^44^Ca | ^66^Zn, ^77^Se | ^111^Cd, ^125^Te | ^208^Pb |
| --- | --- | --- | --- | --- |
| **Dwell Time (ms)** | 100 | 100 | 100 | 100 |
| **RPa** | 0 | 0 | 0 | 0 |
| **RPq** | 0.25 | 0.25 | 0.25 | 0.25 |
| **Calibration (µg/L)** | 0.1 - 5000 | 0.1 - 5000 | 0.1 - 5000 | 0.1 - 5000 |
| **Replicates Per Sample** | 5 | 5 | 5 | 5 |
| **LOD (µg/L)** | See data | See data | See data | See data |
| **LOQ (µg/L)** | See data | See data | See data | See data |
| **Internal Standard (µg/L)** | ^45^Sc | ^71^Ga | ^89^Y | ^191^Ir |

**Supplementary Table 3:** Experimental parameters and data acquisition parameters used for LA-ICP-MS imaging of Pb in the earthworm brain at 4-micron pixel resolution.

|  | **Brain** | **NIST 612 Scans** |
| --- | --- | --- |
| **Teledyne Photon Machines Analyte Excite** |  |  |
| Energy density (J cm^-2^) | 0.8 | 2.0 |
| Repetition rate (Hz) | 75 | 75 |
| Scan speed (µm s^-1^) | 62.5 | 500 |
| Beam waist diameter (µm) | 5 (Circle) | 40 (Circle) |
| Scanning Mode | Fixed Dosage | Fixed Dosage |
| Scanning Direction | Uni-directional | Uni-directional |
| Effective Dosage (shots per position) | 6 | 6 |
| He Carrier gas flow rate (L min ^-1^) | 0.5 | 0.5 |
| **Thermo Fisher Scientific iCAP TQ ICP-MS** |  |  |
| RF power (W) | 1550 | 1550 |
| Ar plasma gas flow rate (L min ^-1^) | 14 | 14 |
| Ar auxiliary gas flow rate (L min ^-1^) | 0.8 | 0.8 |
| Nebuliser gas flow rate (L min ^-1^) | 1.03 | 1.03 |
| He gas flow rate (L min^-1^) | 3.25 | 3.25 |
| TQ-O_2_ gas flow rate (L min^-1^) | 0.6 | 0.6 |
| Acquired m/z ratios (amu) | ^208^Pb | ^208^Pb |
| Respective dwell times (ms) | 80 | 80 |
| Total scan cycle time (ms) | 80 | 80 |

**Supplementary Table 4:** Experimental parameters and data acquisition parameters used for LA-ICP-MS imaging of Pb, Zn, and P in the earthworm head and gut sections at 15-micron pixel resolution.

|  | **Gut and Brain** | **NIST 612 Scans** |
| --- | --- | --- |
| **Teledyne Photon Machines Analyte Excite** |  |  |
| Energy density (J cm^-2^) | 0.8 | 2.0 |
| Repetition rate (Hz) | 60 | 60 |
| Scan speed (µm s^-1^) | 150 | 400 |
| Beam waist diameter (µm) | 15 (Circle) | 40 (Circle) |
| Scanning Mode | Fixed Dosage | Fixed Dosage |
| Scanning Direction | Uni-directional | Uni-directional |
| Effective Dosage (shots per position) | 6 | 6 |
| He Carrier gas flow rate (L min ^-1^) | 0.5 | 0.5 |
| **Thermo Fisher Scientific iCAP TQ ICP-MS** |  |  |
| RF power (W) | 1550 | 1550 |
| Ar plasma gas flow rate (L min ^-1^) | 14 | 14 |
| Ar auxiliary gas flow rate (L min ^-1^) | 0.8 | 0.8 |
| Nebuliser gas flow rate (L min ^-1^) | 1.03 | 1.03 |
| He gas flow rate (L min^-1^) | 3.25 | 3.25 |
| TQ-O_2_ gas flow rate (L min^-1^) | 0.6 | 0.6 |
| Acquired m/z ratios (amu) | ^31^P, ^66^Zn, ^208^Pb | ^31^P, ^66^Zn, ^208^Pb |
| Respective dwell times (ms) | 5, 55, 11 | 5, 55, 11 |
| Total scan cycle time (ms) | 100 | 100 |
